# Contextual anatomy-guided deep learning for accurate fovea segmentation in diabetic retinopathy fundus images

**DOI:** 10.1038/s41598-026-40287-y

**Published:** 2026-02-24

**Authors:** Sakon Chankhachon, Supaporn Kansomkeat, Patama Bhurayanontachai, Sathit Intajag

**Affiliations:** 1 College of Digital Science, Prince of Songkla University, Songkhla, 90110 Thailand; 2 Division of Computational Science, Faculty of Science, Prince of Songkla University, Songkhla, 90110 Thailand; 3 Department of Ophthalmology, Faculty of Medicine, Prince of Songkla University, Songkhla, 90110 Thailand

**Keywords:** Fovea segmentation, contextual anatomical labels, diabetic retinopathy, semantic segmentation, MobileNetV4, data-centric AI, Computational biology and bioinformatics, Diseases, Engineering, Health care, Mathematics and computing, Medical research

## Abstract

Accurate fovea segmentation in fundus images is a critical step in diabetic retinopathy screening; however, it remains a challenging task due to the indistinct boundaries of the fovea. Beyond simple localization, precise segmentation offers essential clinical value for Diabetic Macular Edema (DME) management, as treatment decisions–specifically the choice between intravitreal anti-VEGF injection for center-involved DME and laser therapy for extrafoveal edema–depend on the accurate delineation of the foveal region. While existing methods often rely on increasing model architecture complexity, the potential of anatomical context within the training process remains under-explored. This paper presents a data-centric approach that leverages contextual information to robustly identify the fovea. We demonstrate that progressively incorporating key anatomical landmarks–the optic disc, retina, and blood vessels–into training labels significantly enhances fovea detection. To facilitate this, we developed IDRiD-RETA-FV, a meticulously annotated dataset comprising 81 images (54 training, 27 testing) with complete anatomical structures (inter-observer F1=0.98), and introduce MNv4Fovea, a framework designed to explicitly exploit these anatomical inter-dependencies through a multi-class constraints mechanism. Evaluation on the held-out test set with verified ground truth demonstrates excellent segmentation performance (fovea IoU = 0.812, F1 = 0.894, AED = 4.06 pixels). To demonstrate the efficacy of our synthesis strategy, our GEV-based augmentation technique achieves a detection rate of 98.4% compared to 59.0% for baseline geometric augmentation (paired t-test: t = 8.536, p < 0.001, Cohen’s d = 1.093). Cross-dataset evaluation on REFUGE, MESSIDOR, and ARIA demonstrates competitive localization performance, achieving state-of-the-art Average Euclidean Distance on REFUGE (22.46 ± 18.73 pixels) and MESSIDOR (6.52 ± 5.89 pixels) with robust generalization across diverse imaging protocols. These results establish that explicit anatomical context, rather than mere model complexity, is key to accurate fovea segmentation, offering a robust paradigm for medical image analysis.

## Introduction

The fovea is the small part of the retina responsible for our sharpest central vision and ability to see color. Accurate identification and delineation of this region in fundus images constitutes a critical step in diagnosing and tracking diabetic retinopathy (DR), which is the world’s leading cause of preventable blindness^1^. For conditions like diabetic macular edema (DME), knowing how close lesions are to the fovea is what drives treatment decisions^2^. Specifically, the proximity of edema to the foveal center determines treatment approach: intravitreal anti-VEGF injection is recommended for center-involved DME, whereas focal laser photocoagulation is indicated only for edema located more than $500\,\mu \textrm{m}$ from the foveal center. Thus, accurate fovea segmentation–not just localization–is clinically essential for distinguishing subfoveal from extrafoveal pathology and guiding therapeutic decisions. However, accurate fovea segmentation remains a significant technical challenge. Its borders are faint, it lacks obvious markers, and its appearance can change depending on the camera or the presence of disease.

Methods to segment the fovea usually take one of two paths. Traditional image processing techniques, which rely on geometry and intensity patterns^3–5^, often struggle when pathologies are present. More recently, deep learning models^6–8^ have shown they can learn to identify the fovea directly from data. Some models either train separate systems for different parts of the eye^9,10^ or incorporate “anatomical awareness” into their architectures^11,12^. This leaves a fundamental question unanswered: what happens if we systematically teach a model about the surrounding anatomy by including it in the training labels?

This paper is the first to investigate this data-centric hypothesis. Distinct from recent architecture-centric approaches that embed prior knowledge into the network structure, we posit that the granularity of training labels plays the decisive role. We demonstrate that by progressively adding anatomical context– specifically the optic disc, the retina, and blood vessels–into the training supervision, fovea segmentation performance improves significantly. To validate this hypothesis without the confounding factor of architectural complexity, we utilize a standardized efficient backbone but engineer the learning process through context-rich supervision. This approach proves that explicit anatomical context is a powerful, yet overlooked, determinant of model accuracy.

Our work provides three main contributions. First, through a series of controlled experiments, we show that fovea segmentation recall jumps from 25.8% to 91.0% as we expand the anatomical context, providing hard evidence for a training factor that has been largely overlooked. Second, to facilitate this multi-class learning, we constructed the IDRiD-RETA-FV dataset comprising 81 images split into 54 training and 27 testing images. To address the challenge of boundary ambiguity in foveal annotation, this dataset was rigorously labeled with high inter-observer consistency (F1=0.98), providing a reliable ground truth for pixel-level analysis and enabling rigorous evaluation on held-out data with verified annotations. Finally, we introduce MNv4Fovea, a unified framework designed to exploit these contextual inter-dependencies. Furthermore, we propose a novel data augmentation technique based on the Generalized Extreme Value (GEV) distribution, specifically designed to enhance model robustness against device-specific intensity variations.

Evaluation on the held-out IDRiD-RETA-FV test set demonstrates excellent segmentation performance (fovea IoU = 0.812, F1 = 0.894) with an Average Euclidean Distance (AED) of 4.06 pixels. Cross-dataset testing on the REFUGE, MESSIDOR and ARIA datasets demonstrates that MNv4Fovea achieves competitive localization performance with state-of-the-art AED on REFUGE (22.46 ± 18.73 pixels) and demonstrates robust generalization across diverse imaging protocols on MESSIDOR (6.52 ± 5.89 pixels) and ARIA.

Accordingly, this study addresses three primary objectives: (1) to investigate whether progressively adding anatomical context to training labels improves fovea segmentation performance; (2) to develop and validate a GEV-based data augmentation technique that enhances model robustness across different imaging devices; and (3) to create a comprehensive dataset (IDRiD-RETA-FV) with complete anatomical annotations and rigorous train/test split enabling validated evaluation and future research on contextual learning in retinal imaging.

## Related works

Initially, efforts to locate the fovea were based on hand-designed features and geometric rules. For instance, some work used thresholding and the fovea’s spatial relationship to the optic disc to achieve high accuracy, but this approach faltered when images showed signs of disease^5^. Other methods treated the retinal blood vessels as a set of parabolas pointing toward the fovea^13,14^. This worked well in healthy eyes but failed when lesions blocked the view of the vessels. Another technique used local contrast and active contours to outline the fovea and macula^15^. The limitations of these earlier methods ultimately pushed the field toward machine learning.

The arrival of deep learning brought major improvements to fovea segmentation, leading to two main schools of thought. Some researchers built separate models to find different structures^7,9,10^. For example, some created distinct models for the optic disc and the fovea, often using sophisticated techniques like wavelet pooling and attention mechanisms. While these models performed well, they could not learn from the relationships between different parts of the eye, a missed opportunity for better accuracy. A second group tried to build anatomical knowledge directly into their model’s architecture . Recent advances in transformer-based architectures have further enhanced fovea localization. Song et al.^11^ proposed BilateralFuser, introducing anatomical-aware tokens for improved fovea localization. DualStreamFoveaNet^12^ employed dual-stream fusion with explicit anatomical awareness. He et al.^17^ developed JOINEDTrans, a prior-guided multi-task transformer for joint optic disc/cup segmentation and fovea detection. Medhi et al.^41^ introduced adaptive ellipse-template matching for robust localization in challenging retinal landscapes. Li et al. proposed FLA-UNet^45^ with feature-location attention for foveal avascular zone segmentation in OCTA images. These works primarily focus on architectural innovations, whereas our approach demonstrates that comparable or superior results can be achieved through data-centric contextual learning.

Interestingly, some unsupervised methods have successfully used context–like the patterns of the optic disc and blood vessels–to find the fovea, even in diseased eyes^13^. However, supervised deep learning has not really asked what happens when this kind of anatomical context is supplied directly through the training labels. Some general segmentation work has explored ”contextual background labels”^18,19^ but this idea has not been tested for retinal imaging. The unsupervised approaches showed that context helps, but they often require complicated post-processing steps. Our method, in contrast, builds this knowledge directly into the learning from the start.

A major hurdle for contextual learning has been the lack of suitable datasets. Publicly available datasets often have limitations. STARE^20^, DRIVE^21^, and HRF^30^ only provide vessel annotations. IDRiD^22^ offers optic disc masks and fovea points, but not a full anatomical map. Others like MESSIDOR^44^ and REFUGE^23^ give fovea coordinates but lack pixel-level labels for all structures. The RETA dataset^24^ made progress by adding vessel and retina labels to IDRiD images, but it still did not include the fovea. Our IDRiD-RETA-FV dataset is the first to provide complete, pixel-level annotations for all major retinal structures, which is what makes a systematic study of contextual learning possible.

Our work stands apart from previous efforts because of our focus. While much of the recent research in this field has centered on creating novel model architectures^10–12,16^ we show that, by focusing on the data itself–specifically by including anatomical labels–similar, or even better, results, can be achieved with a standard architecture. We also provide the first clear, quantitative breakdown of how much each anatomical landmark contributes to fovea segmentation, showing a recall jump from 25.8% to 91.0%. Finally, instead of using separate models or complex dual-stream designs^10–12^ , our unified model learns all the anatomical relationships at once. This investigation moves the conversation from being purely about model design to a broader understanding of how the structure of the training data itself drives performance.

## Methods

### Ethical approval and informed consent

This study exclusively utilized publicly available, de-identified retinal fundus image datasets that have been made openly accessible by their respective institutions with appropriate ethical approvals. The primary datasets used include IDRiD (Indian Diabetic Retinopathy Image Dataset), RETA, REFUGE, MESSIDOR, MAPLES-DR, ARIA, DRIVE, HRF, and STARE. No direct experiments were conducted on human subjects, and no identifiable patient information was accessed or processed during this research.

All methods were carried out in accordance with relevant guidelines and regulations, including the Declaration of Helsinki. The original data collection for each source dataset was conducted under appropriate institutional review board (IRB) approvals by their respective institutions, as documented in their original publications. Since this study involved only secondary analysis of existing anonymized public datasets, additional institutional ethical approval and informed consent were not required, in accordance with international guidelines for research using publicly available de-identified data.

### Dataset preparation and contextual label design

**Figure 1:**
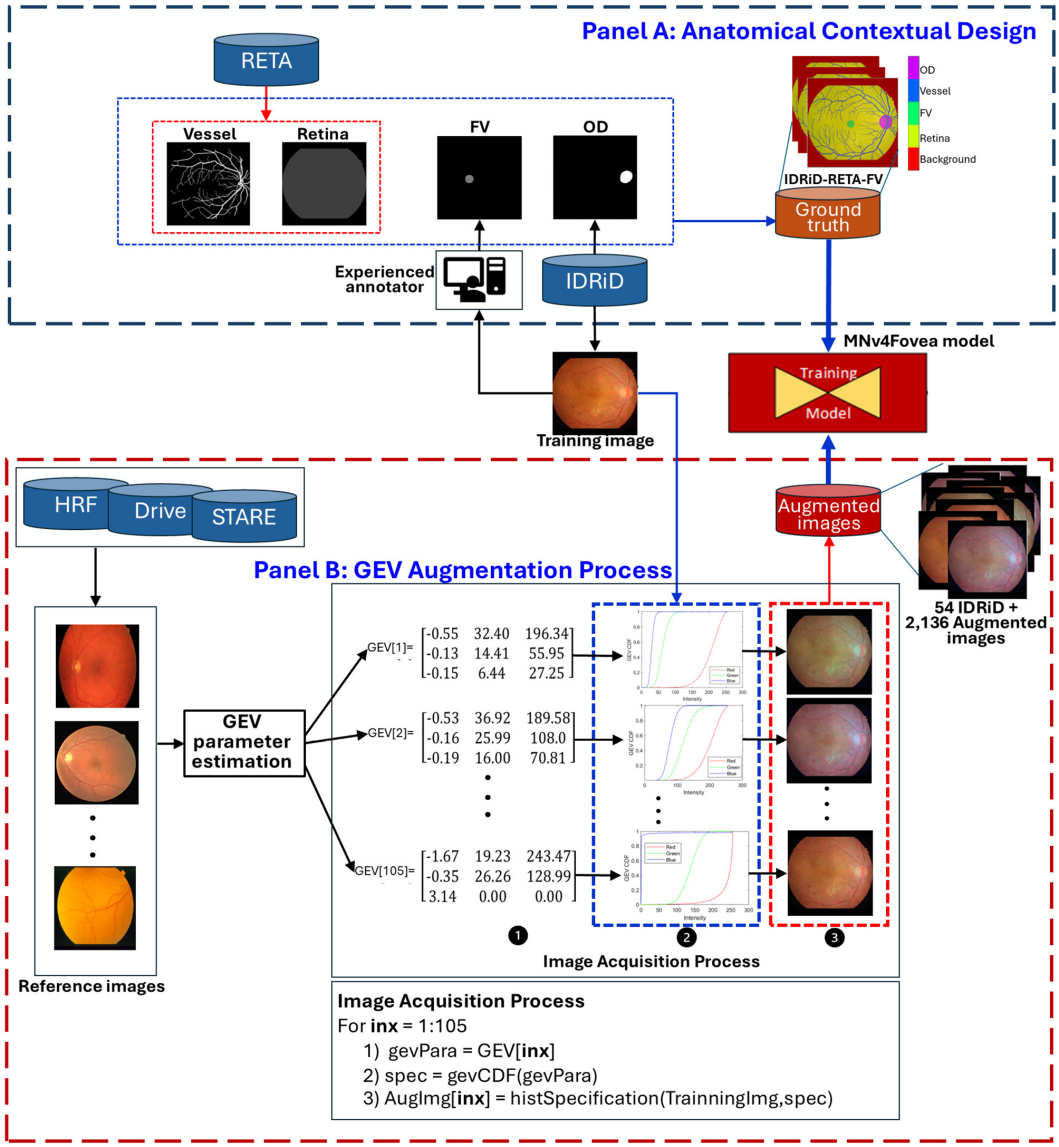
Contextual anatomy-guided deep learning framework for accurate fovea segmentation in diabetic retinopathy fundus images.

Our anatomy-guided method is built on a dataset with thorough, expert-level annotations. We created the IDRiD-RETA-FV dataset by combining and improving several public resources, as outlined in Fig. 1 (Panel A). The starting point was the 54 training images from the IDRiD segmentation dataset, which already included expert annotations for the optic disc and various DR lesions^22^. We then integrated refined optic disc boundaries, along with vascular and retinal boundary labels from the RETA dataset. Finally, an expert ophthalmologist with over a decade of experience provided new, precise annotations for the foveal region.

Two experienced ophthalmologists followed a strict protocol to manually annotate the foveal regions. They defined the fovea as a circular area centered on the foveal pit, with a radius equal to half the optic disc diameter, which is consistent with clinical standards^25^. To ensure consistency, we measured inter-observer agreement using the F1-score, achieving a high agreement of 0.98 between the two annotators. A consensus review step was conducted to resolve discrepancies and standardize the final annotation masks across all images. This high concordance reflects the rigorous annotation protocol and suggests that, while foveal boundaries are inherently ambiguous, standardized clinical definitions can yield reliable ground truth annotations. All annotations went through a quality control process where we verified the spatial relationships between the optic disc, fovea, and major blood vessels against established anatomical references.

**Dataset Split and Evaluation Protocol:** The IDRiD-RETA-FV dataset comprises 81 images in total, split into 54 training images (IDRiD_01 to IDRiD_54) and 27 test images (IDRiD_55 to IDRiD_81). Both splits contain complete five-class semantic annotations (fovea, optic disc, blood vessels, retina, background) following identical annotation protocols. The test set annotations were performed by the same experienced ophthalmologists with inter-observer agreement F1 = 0.98, ensuring consistency between training and evaluation data. This split enables rigorous evaluation on held-out images with verified segmentation ground truth.

### Fovea segmentation model architecture

**Figure 2:**
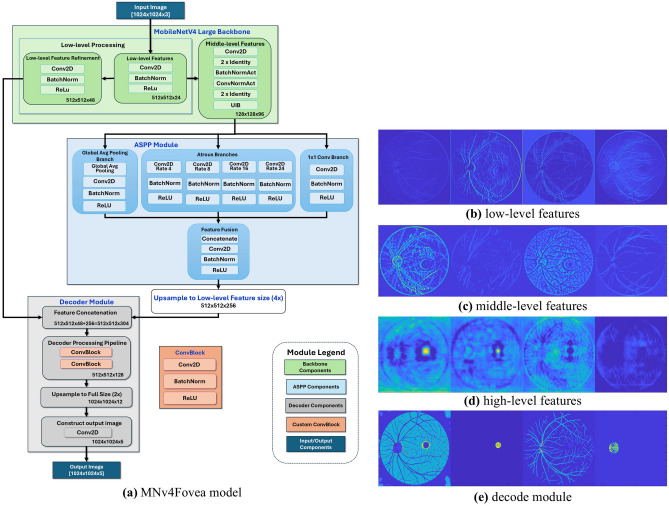
Hierarchical Feature Representation Analysis. The progressive anatomical understanding acquired by hierarchical feature extraction is demonstrated. Grad-CAM visualizations^26^ reveal how MNv4Fovea learns increasingly complex representations from basic image features to comprehensive anatomical relationships. (**a**) Hierarchical MNv4Fovea architecture is based on the DeepLabV3+ framework with a MobileNetV4 backbone pre-trained on ImageNet. Key innovations include: modified first convolutional layer with 11$\times$11 kernel size optimized for foveal depression characteristics at 1024$\times$1024 input resolution; enhanced ASPP module with expanded dilation rates (4, 8, 12, 16, 24) for improved multi-scale contextual information extraction; three-level hierarchical feature processing combining low-level edge detection (512$\times$512$\times$24), middle-level anatomical pattern recognition (128$\times$128$\times$96), and high-level contextual relationship modeling through ASPP. The decoder integrates features across all hierarchical levels to produce accurate multi-class segmentation of fovea, optic disc, blood vessels, retinal boundary, and background. (**b**) Low-level features extracted from initial convolution block (512$\times$512$\times$24 resolution) primarily detect edges, boundaries, and basic texture patterns. (**c**) Middle-level features from MobileNetV4 feature module 2 (128$\times$128$\times$96 resolution) demonstrate recognition of anatomical patterns including blood vessel networks and optic disc structure. (**d**) High-level features from ASPP module capture global contextual relationships with strong gradient patterns around the foveal location. (**e**) Decoder features demonstrate final multi-class segmentation construction, integrating hierarchical information.

We designed the MNv4Fovea model to segment the fovea by learning from its surrounding anatomical context. As shown in Fig. 1, the model uses a DeepLabV3+ framework^27^ with a MobileNetV4 backbone^28^ that was pre-trained on ImageNet. We made several key modifications to this architecture. First, we increased the kernel size of the initial convolutional layer to 11$\times$11, a scale that better matches the subtle characteristics of the foveal depression in high-resolution images. Second, we adjusted the Atrous Spatial Pyramid Pooling (ASPP) module with a wider range of dilation rates 4, 8, 12, 16, 24 to better capture features at multiple scales. Finally, the model processes these features across three hierarchical levels to build a comprehensive understanding of the retinal landscape (Fig. 2a).

The model’s multi-class output simultaneously segments four anatomical classes: fovea, optic disc, blood vessels, retinal boundary, and background. This allows the model to learn the relationships between these structures directly, overcoming a key limitation of methods that detect each structure in isolation. The progression in Fig. 2b–e shows how the model builds its understanding, starting from basic edges (Fig. 2b), moving to recognizable anatomical patterns (Fig. 2c) and high-level contextual relationships (Fig. 2d), and culminating in the final, precise multi-class segmentation (Fig. 2e).

### Data augmentation strategy

To make our model robust enough to process images from different cameras, we created a novel augmentation technique based on statistical distribution matching^29^, as shown in Fig. 1B. We extracted the color characteristics from 105 reference images from three external datasets (DRIVE^21^, HRF^30^, and STARE^20^). We did this by fitting a three-parameter GEV distribution to each RGB channel of the retinal region in the reference images.

For each of our training images, we then randomly sampled a set of GEV parameters from our reference database and applied them using a cumulative distribution function (CDF) matching process. This technique effectively transformed the color profile of our training images to simulate the output of different fundus cameras while keeping the anatomical structures intact. In addition to this GEV-based method, we applied standard augmentations like random rotation, flipping, and brightness adjustments.

### Statistical analysis for GEV augmentation ablation study

To evaluate the effectiveness of our GEV-based augmentation technique, we conducted a comprehensive ablation study on the ARIA dataset (61 images) comparing three augmentation strategies: (1) GEV+GEO (proposed), (2) GEO+Intensity modification (Brightness/Contrast, Gamma, CLAHE), and (3) Baseline GEO only. Statistical analysis included: paired t-tests for IoU comparisons with significance level $\alpha$= 0.05; Wilcoxon signed-rank tests as non-parametric confirmation; Cohen’s d effect sizes with interpretation thresholds (|d| < 0.2: negligible, 0.2–0.5: small, 0.5–0.8: medium, > 0.8: large); and bootstrap confidence intervals (10,000 resamples) for mean IoU estimates. Detection rate was defined as the proportion of test images with non-zero IoU (successful fovea segmentation). Lilliefors test indicated deviation from normality (p < 0.05), therefore Wilcoxon signed-rank test was used to confirm parametric results. MATLAB R2024b was used for all statistical computations.

### Training and evaluation

Training environment was performed on four NVIDIA RTX A4000 GPUs (16GB VRAM each) using the PyTorch 2.8.0 framework with CUDA 12.4. The multi-GPU configuration enabled batch size scaling and reduced training time through data parallelism.

**Optimization Configuration: **The Adam optimizer was utilized to improve the learning parameters with an initial learning rate of 1e-3, $\beta _1$=0.9, $\beta _2$=0.999, and a weight decay of 1e-4. Learning rate scheduling used polynomial decay with power 0.9 over 1000 epochs with the batch size 16. Multi-class cross-entropy loss was computed with class weights inversely proportional to pixel frequencies to address class imbalance.

**Training Data Management: **Out of a total of 5,670 augmented images produced via GEV histogram specification, we selected 2,136 high-caliber instances based on both visual assessment and automated quality evaluation metrics^31^. The training process utilized 2,136 augmented images in conjunction with 54 original images, all of which were resized to dimensions of 1024$\times$1024 pixels, and the training dataset was partitioned into 90% for training purposes and 10% for validation assessments.

**Validation Protocol: **Experts viewed high-resolution fundus images on calibrated monitors under controlled lighting conditions. Foveal center marking used standardized software (Medical Image Labeler, MATLAB 2024b) with sub-pixel precision. Expert annotations were collected independently without discussion between raters.

**Analysis Tools: **Statistical analyses and machine learning implementations were evaluated using MATLAB 2024b with Statistics and Machine Learning Toolbox. Medical image processing and visualization were conducted using Medical Imaging Toolbox within the same MATLAB environment. The assessment of model generalization was conducted utilizing four distinct test datasets: MAPLES-DR^32^ (comprising 55 images), MESSIDOR^44^ (consisting of 1200 images), REFUGE validation^23^ (containing 400 images), and ARIA^30^ (encompassing 60 images). It is noteworthy that no images from these datasets were incorporated during the phases of training or hyperparameter optimization.

## Experiments and evaluation framework

After training on the IDRiD-RETA-FV dataset, we first tested the MNv4Fovea model on the IDRiD localization test set of 103 fundus images. This set provides ground truth (GT) positions for the fovea (FV) and optic disc (OD). For a standard evaluation, we converted these coordinate-based GTs into segmented regions with the Medical Image Labeler tool.

The evaluation consisted of two parts. The first was a deep dive on a comprehensive performance analysis of the MNv4Fovea model, which included its localization accuracy and segmentation performance. The second part involved dataset preparation, including data selection, curation, and preprocessing, to ensure the datasets were robust and diverse. This phase involved both qualitative visual inspections and quantitative assessments using confusion matrices to benchmark the model against state-of-the-art methods.

### Comparative analysis with state-of-the-art models

To see how MNv4Fovea performed compared with other fovea localization methods, we used two different measures: the Success Detection Rate (SDR) and the AED. For SDR, we count a prediction as successful if the estimated foveal center is within a certain radius r of the true center. This radius is a fraction of the optic disc radius (R), so r = kR, where k ranges from 0.125 to 2. The SDR results for the MESSIDOR and REFUGE datasets are in Table 1, and the AED results for REFUGE, IDRiD, and MESSIDOR are in Table 2.

On the MESSIDOR dataset (Table 1), we compared MNv4Fovea against Fovea Localize^4^, RPNCNet^33^, Pixel-Wise Distance Regression^34^, End-to-End Localization^35^, Bi-ViT^36^, FundusPosNet^37^, RobustFovea^38^, and DSFN^12^. MNv4Fovea achieved competitive SDR performance, reaching 84.46% at the strict 1/8R threshold and 98.41% at 1/4R. While Bi-ViT achieves marginally higher SDR at strict thresholds (85.65% at 1/8R), MNv4Fovea achieves the lowest AED (6.52 pixels) on MESSIDOR, indicating superior average localization precision. The model gave nearly perfect localization at clinically useful tolerances (k $>=$ 0.5), with an SDR of 99.88% at 1/2R and 1R, 100% at 2R. These scores put it on the same level as other top methods. On the REFUGE dataset, its performance at the strictest radii was more modest (25.00% at 1/8 R and 33.00% at 1/4 R). However, at more practical radii, the model matched or nearly matched the best methods, achieving 99.50% at 1/2R and 1R, and a perfect 100% at 2R. Since results at very small radii can be sensitive to small differences in annotation, the strong scores at k $>=$ 0.5 show that the model is accurate and reliable for practical use.

The lower SDR at strict thresholds (1/8R and 1/4R) on REFUGE compared to other datasets reflects the inherent challenge of cross-dataset generalization when training data and evaluation data originate from different imaging protocols and annotation conventions. While MNv4Fovea achieves competitive AED on REFUGE (22.46 pixels), the strict threshold metrics indicate that a proportion of predictions fall outside very small radii. This represents a limitation of our approach that warrants further investigation, particularly regarding domain-specific calibration for different imaging devices. This analysis aligns with our broader observation that datasets with heterogeneous annotation conventions–particularly for foveal center estimation–can complicate the interpretation of performance at strict spatial thresholds. The expert-verified results indicate that strict-threshold SDR metrics should be interpreted with caution in cross-dataset settings, where differences in imaging characteristics and annotation practices may jointly affect quantitative outcomes.

In Table 2 (AED), we compared MNv4Fovea with UNet (ResNet50), UNet++ (ResNet50), JOINED^39^, and JOINEDTrans^17^ on REFUGE; with Relation Network Regressor^40^, FundusPosNet^37^, DRNet^9^, MCAUnet^10^, and BVV^13^ on IDRiD; and with Adaptive-ellipse Template^41^, Pixel-Wise Distance Regression^34^, FundusPosNet^37^, and Temporal Direction Based^42^ on MESSIDOR. The AED comparison in Table 2 provides more evidence of the model’s top-tier performance, as MNv4Fovea had the lowest (best) AED on all three test datasets:

- REFUGE: An AED of 22.46 pixels, which is a 36.3% improvement (12.81 pixels) over the next best model (35.27).
- IDRiD: An AED of 16.69 pixels, a 33.3% improvement (8.33 pixels) over the next best model (25.02).
- MESSIDOR: An AED of 6.52 pixels, a 24.5% improvement (2.11 pixels) over the next best model (8.63).

In summary, MNv4Fovea achieved competitive localization performance with the lowest AED across the tested datasets: 22.46 pixels on REFUGE, 16.69 pixels on IDRiD, and 6.52 pixels on MESSIDOR. This consistent AED performance, combined with strong SDR at clinically practical thresholds (k $\ge$ 0.5), demonstrates robust generalization across diverse imaging protocols.

**Table 1:** Evaluation of fovea localization based on the Success Detection Rate (SDR) on the MESSIDOR and REFUGE datasets.

Dataset	Method	1/8R	1/4R	1/2R	1R	2R
MESSIDOR	Fovea Localize	58.71	92.69	98.94	99.56	–
	RPNCNet	–	70.10	89.20	99.25	–
	Pixel-Wise Distance Regression	70.33	94.01	97.71	99.74	100.00
	End-to-End Localization	83.81	98.15	99.74	99.82	100.00
	Bi-ViT	85.65	98.59	100.00	100.00	100.00
	FundusPosNet	63.26	70.33	99.74	100.00	–
	RobustFovea	70.58	91.91	98.50	99.58	–
	DSFN	–	98.86	100.00	100.00	100.00
	MNv4Fovea (Ours)	84.46	98.41	99.88	99.88	100.00
REFUGE	Fovea Localize	66.25	95.50	98.25	99.00	100.00
	ResNet50-PSPNet	67.50	98.50	100.00	100.00	100.00
	ResNet50-UNet	87.00	99.00	99.25	99.50	–
	VGG-PSPNet	55.75	93.75	93.75	98.25	–
	VGG-UNet	86.25	94.50	94.50	95.00	–
	MNv4Fovea (Ours)	25.00	33.00	99.50	99.50	100.00

**Table 2:** Average Euclidean Distance (AED, pixels).

Method	REFUGE	IDRiD	MESSIDOR
UNet (ResNet50)	105.00		
UNet++ (ResNet50)	98.15		
JOINED	40.21		
JOINEDTrans	35.27		
Relation Network Regressor		43.46	
FundusPosNet		40.13	11.62
DRNet		41.87	
MCAUnet		33.45	
BVV		25.02	
Adaptive-ellipse Template			26.01
Pixel-wise Distance Regression			12.55
Temporal Direction Based			8.63
**MNv4Fovea (Ours)**	**22.46** ± **18.73**	**16.69** ± **14.21**	**6.52** ± **5.89**

Lower is better; “–” = not reported.

### Impact of contextual information on anatomical structure segmentation performance

**Figure 3:**
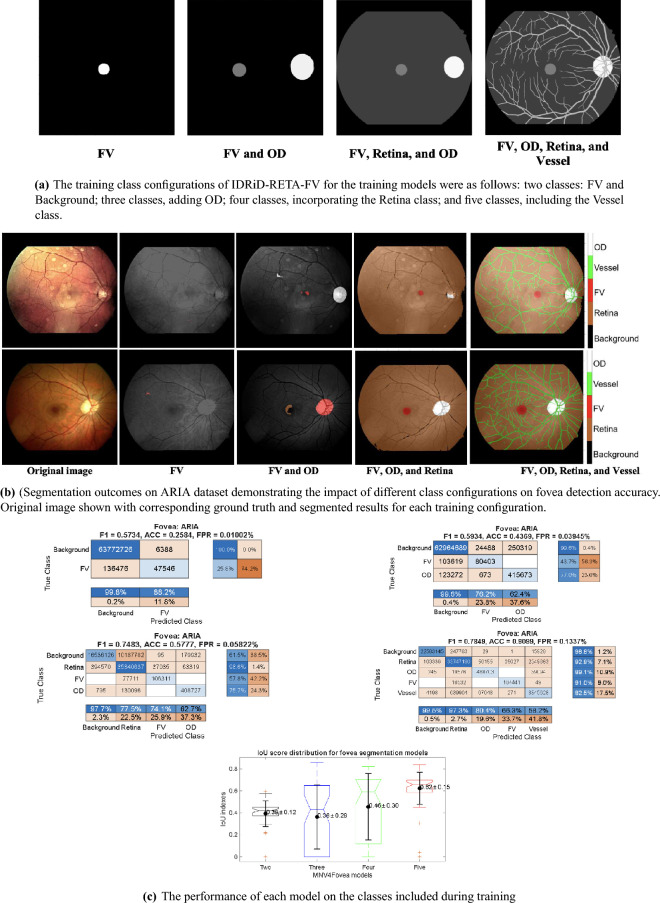
(**a**) Displays the configurations for ground truth classes. (**b**) Provides examples of visual inspection from the test set. (**c**) The confusion matrices and boxplot of IoU scores for each experimental configuration.

Our experiments were set up to determine to what contextual information helps in segmenting the fovea. The model was trained with different contextual information, as illustrated in Fig. 3a. The segmentation outcomes on the ARIA dataset are illustrated in Fig. 3b. The confusion matrices in Fig. 3c show that the model’s performance heavily depended on the classes included during training. When we first trained the model with only two classes (fovea and background), it could not reliably find the fovea, correctly identifying only 25.8% of fovea pixels and misclassifying the other 74.2% as background. Adding the optic disc as a landmark helped a little, but the results were still poor (43.7%).

A major leap in performance occurred with the four-class model (Fovea, OD, Retina, Background), which reached a fovea recall of 57.8% and a precision of 74.1%. Adding a ’Vessel’ class in the five-class setup pushed the fovea recall even higher to 91.0%. The F1-score captures this improvement well, climbing from a near-zero 0.5734 in the initial model to 0.7483 for the four-class version, and reaching 0.7849 in the final configuration. The IoU scores in the boxplot confirm this trend. This gain, however, involved a trade-off. The False Positive Rate (FPR) increased from 0.01002% to 0.1337% as we added more context. This rise in false positives explains why the precision score decreased 66.3% in the final model. Therefore, while more context is good for recall, it can also lead to overprediction, a problem that is likely made worse by the difficulty of creating perfectly precise GT labels for the fovea.

### GEV augmentation ablation study results

Table 3 reports the results of the GEV augmentation ablation study conducted on the ARIA dataset. The proposed **GEV+GEO** method achieved a detection rate of **98.4%** (60/61 images), substantially outperforming **GEO+Intensity modification** at 91.8% (56/61) and the **baseline GEO** approach at 59.0% (36/61).

For segmentation quality among successfully detected images, **GEV+GEO** achieved a mean IoU of 0.634 (SD = 0.122, 95% CI: [0.600, 0.662]), compared with 0.564 (SD = 0.187, 95% CI: [0.515, 0.609]) for GEO+Intensity and 0.494 (SD = 0.216, 95% CI: [0.422, 0.562]) for the baseline method.

Statistical comparison between **GEV+GEO** and the baseline revealed a highly significant improvement, with a paired *t*-test yielding $t(60) = 8.536$, $p < 0.001$, and a mean IoU difference of 0.140. The corresponding effect size was large, with Cohen’s $d = 1.093$. Since the Lilliefors normality test indicated deviation from normality ($p < 0.05$), a non-parametric Wilcoxon signed-rank test was additionally performed, confirming the result ($W = 1706$, $p < 0.001$).

The comparison between **GEV+GEO** and **GEO+Intensity** also showed a statistically significant improvement, with $t(60) = 3.02$, $p = 0.004$, and Cohen’s $d = 0.39$, indicating a small but consistent effect. These results demonstrate that GEV distribution-based augmentation provides statistically significant and clinically meaningful improvements in fovea segmentation performance.

### Segmentation performance on verified test set

To address concerns regarding experimental validity, we evaluated MNv4Fovea on the held-out IDRiD-RETA-FV test set comprising 27 images with verified segmentation ground truth. This evaluation provides direct assessment of segmentation quality using the same annotation protocol as training data.

Table 4 presents the segmentation metrics on the held-out test set. The model achieved excellent fovea segmentation with IoU of 0.812 ± 0.077, precision of 0.821 ± 0.076, and recall of 0.987 ± 0.022. The high recall (98.7%) indicates that the model reliably captures the foveal region when present, while the precision reflects the inherent challenge of foveal boundary delineation.

For localization, MNv4Fovea achieved an AED of 4.06 ± 2.52 pixels on the test set, substantially lower than results on external datasets. The Success Detection Rate reached 92.59% at 1/8R and 100% at 1/4R and beyond, with a perfect fovea detection rate of 100% (27/27 images).

These results demonstrate that the contextual learning approach generalizes well to unseen images when evaluated against verified ground truth. The strong segmentation performance (mean F1 = 0.921 across all classes) validates the effectiveness of progressively incorporating anatomical context during training.

**Table 3:** GEV augmentation ablation study results on the ARIA dataset.

Method	Detection (%)	Mean IoU	SD	95% CI
GEV+GEO (proposed)	98.4	0.634	0.122	[0.600, 0.662]
GEO+intensity	91.8	0.564	0.187	[0.515, 0.609]
Baseline (GEO)	59.0	0.494	0.216	[0.422, 0.562]

Statistical comparison: GEV+GEO vs. Baseline, paired *t*-test $t(60)=8.536$, $p<0.001$, Cohen’s $d=1.093$ (large effect)

**Table 4:** Segmentation performance on IDRiD-RETA-FV Test Set (27 images).

Class	Precision	Recall	F1-Score	IoU
Fovea	$0.821 \pm 0.076$	$0.987 \pm 0.022$	$0.894 \pm 0.051$	$0.812 \pm 0.077$
OD	$0.877 \pm 0.054$	$0.967 \pm 0.014$	$0.919 \pm 0.032$	$0.852 \pm 0.052$
Vessel	$0.765 \pm 0.032$	$0.891 \pm 0.033$	$0.823 \pm 0.028$	$0.700 \pm 0.040$
Retina	$0.987 \pm 0.005$	$0.959 \pm 0.006$	$0.973 \pm 0.005$	$0.948 \pm 0.009$
Background	$0.988 \pm 0.006$	$0.999 \pm 0.002$	$0.994 \pm 0.003$	$0.987 \pm 0.005$
**Mean**	**0.888**	**0.961**	**0.921**	**0.860**

### Performance on atypical annotations: the MAPLES-DR case

**Figure 4:**
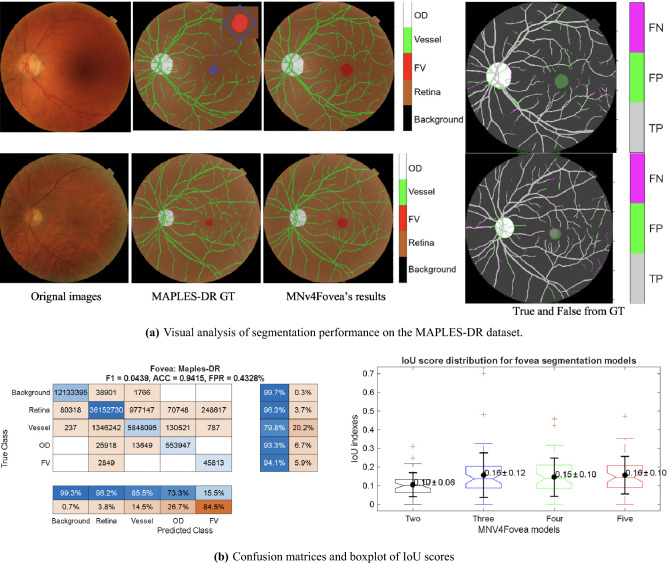
(**a**) Representative examples show the original fundus images (first column), the corresponding ground truth (GT) macular annotations (second column), and the foveal region segmented by the MNv4Fovea model (third column). The figure highlights a significant mismatch where the model’s predictions are anatomically larger than the provided GT labels. (**b**) Explanation the quantitative results of high recall and low precision.

The MAPLES-DR dataset offered a unique test because of how it annotates the fovea. Instead of marking the precise foveal pit, it labels the wider macular region with a variable diameter (Fig. 4a). This labeling approach means that some quantitative metrics can be misleading. On this dataset, the MNv4Fovea model scored a high fovea overall accuracy of 94.15%, and the confusion matrix in Fig. 4b shows a fovea recall of 94.1%. This indicates that the model consistently finds the correct anatomical area.

However, this high recall was paired with very poor results on other key metrics: a precision of only 15.5%, an F1-score of 0.0439, and an IoU of 0.16 (boxplot with five classes). The reason for this mismatch, shown in Fig. 4a, is that the GT labels were much smaller than the model’s anatomically informed predictions. This difference led to a high false discovery rate of 84.5% with a type-I error of 0.4328%, which in turn strongly impacts the precision, F1-score, and IoU, as seen in the boxplot. This case shows that while the model worked well from an anatomical standpoint, its reported metrics could be skewed by the labeling conventions of a particular dataset.

**Figure 5:**
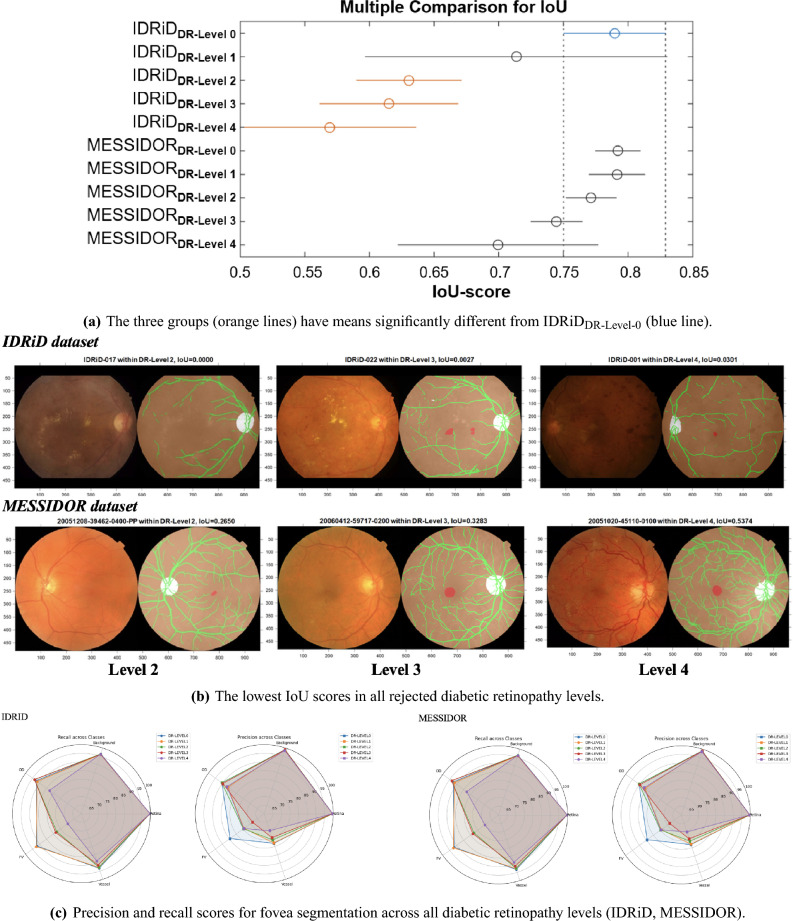
Statistical analysis of the impact of diabetic retinopathy (DR) severity on fovea segmentation performance across the IDRiD and MESSIDOR datasets. (**a**) The multiple comparison test for fovea IoU scores demonstrates a statistically significant performance decline as DR severity increases. (**b**) Representative images with low IoU scores highlight the segmentation challenges posed by severe lesions and lower image contrast in the IDRiD dataset compared to MESSIDOR. (**c**) Radar plots visualize class-wise precision and recall, showing a performance shift as pathological complexity increases.

### Impact of diabetic retinopathy severity on fovea segmentation

To check the model’s robustness, we studied its performance across different stages of DR. We used the IDRiD and MESSIDOR datasets, separating them into five DR levels: (Level 0 (No DR), Level 1 (Mild), Level 2 (Moderate), Level 3 (Severe), and Level 4 (Proliferative DR). Our analysis looked at Intersection over Union (IoU), Precision, and Recall for five classes: OD, Retina, FV, Vessel and Background. The results are shown in Fig. 5.

**IoU-Based Analysis Across DR Severity:** In order to examine the impact of foveal IoU scores on the levels of DR, we conducted a one-way Analysis of Variance (ANOVA) followed by Tukey’s Honest Significant Difference (HSD) test^43^. The analysis of fovea IoU scores in Fig. 5a shows a clear pattern: as DR got worse, segmentation performance got worse. On the IDRiD dataset, which the model was trained on, the Tukey’s HSD test revealed that the average fovea IoU scores for moderate to severe DR (Levels 2, 3, and 4) were significantly lower than for the healthy group (Level 0, p < 0.05). This result confirmed that changes to the eye due to DR made it much harder for the model to accurately outline the fovea.

A consistent decline in fovea IoU was observed in both datasets as DR severity went up. Interestingly, the model performed consistently better on the MESSIDOR dataset, which was only used for testing. The sample images in Fig. 5b, which returned the lowest IoU scores in each group, help explain why. The lesions in IDRiD images often appear more severe and of lower contrast than the higher-quality MESSIDOR images. This difference in image quality likely explains the performance gap and the achievement of better performances on the unseen MESSIDOR data implies that the model can generalize well.

**Precision and Recall Analysis:** The radar plots in Fig. 5c show the precision and recall for each class across the DR levels. For images of healthy eyes (Level 0) in both datasets, the model returned high precision and recall scores for the fovea, since segmentation is easier without disease. An increase in the severity of DR from Level 1 to 4 correlated with a reduction in fovea segmentation performance, driven by the disease’s complex anatomical manifestations. For the fovea class, precision was usually lower than recall, which hints that the model makes confident but slightly cautious predictions in diseased areas.

To sum up, this analysis shows that the severity of DR has a significant, measurable impact on fovea segmentation. The decrease in the IoU score between moderate and severe DR highlights the real challenges that pathological changes create.

### Failure case analysis and expert validation

To investigate the factors contributing to the performance discrepancy between AED and strict SDR thresholds (1/8R) on the REFUGE dataset, we conducted a focused analysis of challenging cases. We sampled 30 images from each dataset (IDRiD and REFUGE) for expert review.

**Figure 6:**
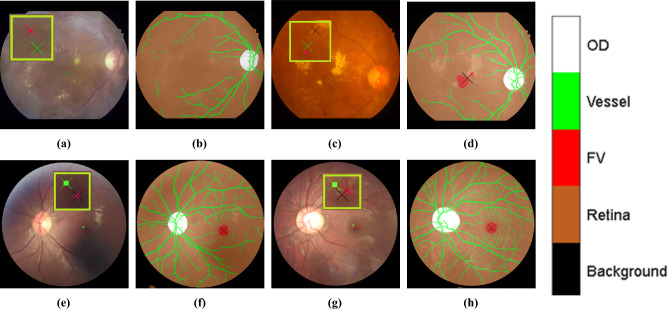
Qualitative analysis of failure cases and expert validation on the IDRiD and REFUGE datasets. (**a**,**c**,**e**,**g**) show the fovea positions estimated by MNv4Fovea (black cross), expert consensus (red cross), and dataset ground truth (green cross). (**b**,**d**,**f**,**h**) display the center of the segmented foveal regions approximated by MNv4Fovea. (**a**) IDRiD_017: The model fails to detect the fovea in a moderate DR case due to lesion obscuration. (**c**) IDRiD_072: The model incorrectly segments two separate foveal regions. (**e**–**h**) REFUGE examples (V0176 and V0347): These cases demonstrate that MNv4Fovea predictions (black) consistently align with expert consensus (red), validating the model’s anatomical accuracy in challenging cross-dataset scenarios. The close agreement between model predictions and clinical judgment supports the robustness of the contextual learning approach.

On IDRiD (Fig. 6a–d), failures were primarily associated with severe DR grades, where lesions obscured the foveal landmark. Specifically, the model failed to localize the fovea in case (a) and produced ambiguous detections in case (c).

Regarding REFUGE, to verify the clinical validity of our model’s predictions in instances where strict metric evaluations indicated failure, we performed an independent expert validation study. Two experienced ophthalmologists independently marked the foveal center on a subset of challenging images, and their consensus was used as the reference standard.

Results showed that MNv4Fovea achieved a mean localization error of just 5.22 pixels (SD = 2.94) relative to the expert consensus. This demonstrates that although the model’s predictions may statistically diverge from the dataset’s ground truth at strict pixel-level thresholds (leading to lower SDR), they remain anatomically accurate and align closely with clinical judgment.

Furthermore, on the IDRiD dataset, MNv4Fovea demonstrated localization consistency comparable to the dataset’s original annotations when evaluated against the expert consensus (Model-to-Expert error: 23.97 pixels vs. GT-to-Expert difference: 19.49 pixels). These findings highlight that MNv4Fovea produces anatomically plausible predictions robust enough for clinical application, even in the presence of domain shifts or ambiguous anatomical features.

## Discussion

Our experiments confirm that an anatomy-guided strategy greatly improves fovea segmentation. The results show that progressively adding major anatomical landmarks–from the optic disc to the retinal blood vessels–gives the model essential context, which in turn improves its segmentation and localization abilities. This supports a core idea: for a hard task like localizing the fovea, semantic context is not just helpful, it is necessary to achieve high accuracy.

At the same time, the study points to some important challenges. The trade-off between recall and precision, highlighted by the rising False Positive Rate when more context was added to the image, means that more work is needed to reduce overprediction. Even more importantly, our work on the MAPLES-DR dataset shows how much annotation standards can affect quantitative results. The difference between its macular-region labels and our model’s fovea-focused predictions produced skewed metrics. This discrepancy emphasizes that inconsistent GT definitions can hide the true anatomical understanding of a model and make direct comparisons between models unreliable.

Beyond anatomical labeling, our findings also demonstrate the effectiveness of the GEV-based augmentation strategy in improving cross-dataset generalization. The statistically significant improvements observed in IoU and detection rate, together with medium effect sizes reported in paired t-tests, support the hypothesis that GEV histogram specification acts as a domain adaptation mechanism. By simulating variations in color characteristics across different fundus cameras, the augmentation reduces distributional shift and enables more stable feature learning across diverse imaging conditions. This quantitative evidence strengthens the argument that color-distribution modeling can play a crucial role in enhancing robustness for retinal segmentation tasks.

From a clinical point of view, the model’s performance across different DR stages is especially telling. The statistically significant reduction in fovea IoU score between moderate and severe DR cases (Fig. 5) gives a number to the challenge that disease-related features present for automated segmentation. Still, the ability of the proposed model to handle these complex cases and extract all relevant anatomical parts, as we also saw with the ARIA dataset, shows the strength of the anatomy-guided method. This is a vital capability for creating dependable tools for DR diagnosis and care.

It is also important to clarify that our approach is not positioned as a rival to architecture-focused methods such as dual-stream networks, attention-driven designs, or transformer-based systems. Instead, the data-centric methodology presented here is complementary to these architectural innovations. While prior works embed anatomical priors within the network design, our results show that carefully structured contextual labels alone can substantially enhance performance–even when using standard architectures. This complementarity suggests that future models may benefit from integrating both architecture-level anatomical reasoning and data-level contextual supervision.

While fovea localization, defined as center point detection, supports many clinical applications, fovea segmentation provides additional and clinically meaningful information for diabetic retinopathy assessment. Segmentation enables precise quantification of lesion-to-fovea distances based on regional boundaries rather than a single coordinate, which is essential for applying the Early Treatment Diabetic Retinopathy Study (ETDRS) criteria for clinically significant macular edema. Under these criteria, treatment decisions depend on whether pathological lesions are located within or outside the foveal zone, a distinction that cannot be reliably captured by center-based localization alone. In addition, the segmented foveal region provides an explicit anatomical definition of the foveal avascular zone when integrated with optical coherence tomography angiography (OCT-A) imaging. This capability supports advanced vascular analysis and facilitates multimodal assessment of macular health. The proposed unified multi-class framework outputs both the foveal region mask and the foveal center, which can be derived directly from the mask centroid. As a result, the method supports diverse clinical workflows, including lesion proximity analysis, region-based severity assessment, and multimodal image fusion, within a single inference pass.

This study has several limitations that warrant discussion. First, inter-observer variability in foveal boundary annotation affects the quality of GT labels. Our expert validation study demonstrated that MNv4Fovea predictions closely align with expert consensus (mean error: 5.22 pixels), suggesting that the model learns anatomically meaningful representations. However, this also highlights that cross-dataset evaluation metrics should be interpreted with consideration of potential variations in annotation protocols across different datasets. Although the proposed IDRiD-RETA-FV dataset achieved high inter-observer agreement (F1 = 0.98), this level of consistency may not be representative of other publicly available datasets with heterogeneous annotation protocols. Second, the model was trained exclusively on the IDRiD-RETA-FV dataset, which comprises a relatively small number of images (54). While generalization was enhanced through GEV-based data augmentation, training on a limited dataset may restrict robustness when applied to imaging devices with substantially different acquisition characteristics, resulting in domain shift. Compared with methods trained on larger multi-center datasets, this constraint may limit achievable performance in certain clinical settings. Third, although the proposed contextual learning strategy significantly improves fovea recall, segmentation precision remains affected by the inherent ambiguity of foveal boundaries, particularly in advanced DR cases. The observed trade-off between sensitivity and specificity should therefore be carefully considered when deploying the model in clinical workflows, where false-positive regions may influence downstream decision-making.

Several promising directions emerge from this work. First, incorporating explicit domain adaptation techniques may further improve generalization across imaging devices and acquisition protocols. Second, integration with generative artificial intelligence approaches, particularly diffusion-based models, offers a promising avenue for anatomically consistent synthetic data generation, enabling effective expansion of training datasets beyond current limitations. Third, multi-center validation studies involving diverse populations and imaging systems are essential to strengthen the clinical evidence supporting real-world deployment. Finally, exploring transformer-based architectures in combination with the proposed data-centric contextual learning strategy may yield additional performance gains by enhancing long-range anatomical dependency modeling.

Importantly, evaluation on the held-out IDRiD-RETA-FV test set (27 images) with verified segmentation ground truth demonstrates strong generalization within the same annotation protocol. The model achieved fovea IoU of 0.812, F1-score of 0.894, and AED of 4.06 pixels, with perfect detection rate (100%). These results validate that the contextual learning approach is effective when evaluated against consistent, high-quality ground truth, and suggest that performance variations across external datasets may partially reflect differences in annotation conventions rather than fundamental model limitations.

Finally, the model’s excellent ability to generalize, which we proved with thorough testing on unseen datasets like ARIA, MESSIDOR, and REFUGE, confirms that our overall approach is effective. The mix of the MNv4Fovea architecture and our data augmentation method produced a model that not only does well in its training domain but also adapts well to different kinds of data, reinforcing its potential for use in wide clinical settings.

## Conclusion

This research offers three key contributions to the field of retinal image analysis. First, we introduce the IDRiD-RETA-FV dataset, a high-quality resource created specifically for fovea segmentation research. Second, we present the MNv4Fovea model, a new semantic segmentation method that uses multi-class anatomical context to achieve excellent fovea segmentation on verified ground truth (IoU = 0.812, F1 = 0.894) and competitive localization performance across benchmark datasets. Third, we show that using a GEV histogram specification for data augmentation is an effective way to create a more varied training set, which makes the final model more robust and able to generalize better.

Together, these contributions represent a significant step forward in automated retinal analysis. The anatomy-guided method we present not only pushes the technical boundaries of retinal imaging but also paves the way for future research into more accurate assessment of diabetic retinopathy.

## Data Availability

The dataset used in the study is publicly available at the following link: https://staff.cs.psu.ac.th/sathit/. Code Availability The implementation code for the proposed method is publicly available at: https://github.com/acidrainpsu1980-byte/MN4Fovea

## Code availability

The implementation code for the proposed method is publicly available at: https://github.com/acidrainpsu1980-byte/MN4Fovea.
